# Cranial solitary osseous plasmacytoma and subjacent amyloid deposition in a dog

**DOI:** 10.1002/ccr3.3160

**Published:** 2020-08-03

**Authors:** Emili Alcoverro, Daniel Sánchez‐Masián, Emanuele Ricci, Frederike Schiborra, Joanna Morris, Riccardo Finotello

**Affiliations:** ^1^ Department of Small Animal Clinical Science Institute of Veterinary Science University of Liverpool Neston UK; ^2^ Anderson Moores Veterinary Specialists Winchester UK; ^3^ Department of Veterinary Pathology and Public Health Institute of Veterinary Science University of Liverpool Neston UK; ^4^ Small Animal Hospital School of Veterinary Medicine College of Medical Veterinary and Life Sciences University of Glasgow Glasgow UK

**Keywords:** canid < veterinary, neurology, neurosurgery, oncology, veterinary

## Abstract

Solitary osseous plasmacytomas affecting the vertebrae, the zygomatic arch, and ribs occur in dogs. In this report, we describe clinical and imaging features of a solitary osseous plasmacytoma affecting the skull with deposition of amyloid forming a mass‐like lesion. To the authors' knowledge, no similar cases have been reported before.

## Graphical abstract

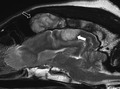

## 1 CASE HISTORY

A 9‐year 1‐month‐old spayed female Labrador Retriever dog weighing 33 kg presented for investigation of three generalized tonic‐clonic epileptic seizures that occurred 1 and 2 months prior to referral; the second and third seizures presented as clusters. Normal interictal periods were documented. The dog was receiving thyroxine supplementation (Thyforon 400 µg tablets; Dechra Veterinary Products) (15 µg/kg PO q24 h) and meloxicam treatment (Loxicom 1.5 mg/mL oral suspension, Norbrook^®^ Laboratories Ltd.) (0.1 mg/kg PO q24 h) for previously diagnosed hypothyroidism and osteoarthritis, respectively. The referring veterinarian had prescribed phenobarbital (Epiphen 60 mg tablets; Vetoquinol UK Ltd) (2 mg/kg PO q12 h) after the last ES. The serum phenobarbital concentration 2 weeks after commencing the anti‐epileptic therapy was 26.8 mg/L (reference range, 25‐30 mg/L for idiopathic epilepsy). Physical examination revealed mild stiff gait on the thoracic limbs, compatible with the aforementioned osteoarthritis. Neurological examination was considered normal, except for mild discomfort upon rostral skull palpation. Based on the patient's age, structural epilepsy was suspected.

Complete blood count revealed mild normocytic, normochromic, and nonregenerative anemia (hematocrit: 30%; reference range, 35%‐55%) and mild thrombocytosis (478 × 10^9^/L; reference range, 150‐400 × 10^9^/L). Serum biochemistry revealed increased alkaline phosphatase (326 U/L; reference range, 0‐100 U/L) and alanine transaminase (67 U/L; reference range, 15‐60 U/L). These clinicopathological changes were most likely secondary to phenobarbital administration. Urinalysis detected no abnormalities. The urine protein: creatinine ratio was normal (0.08; reference range, <0.2).

**FIGURE 1:**
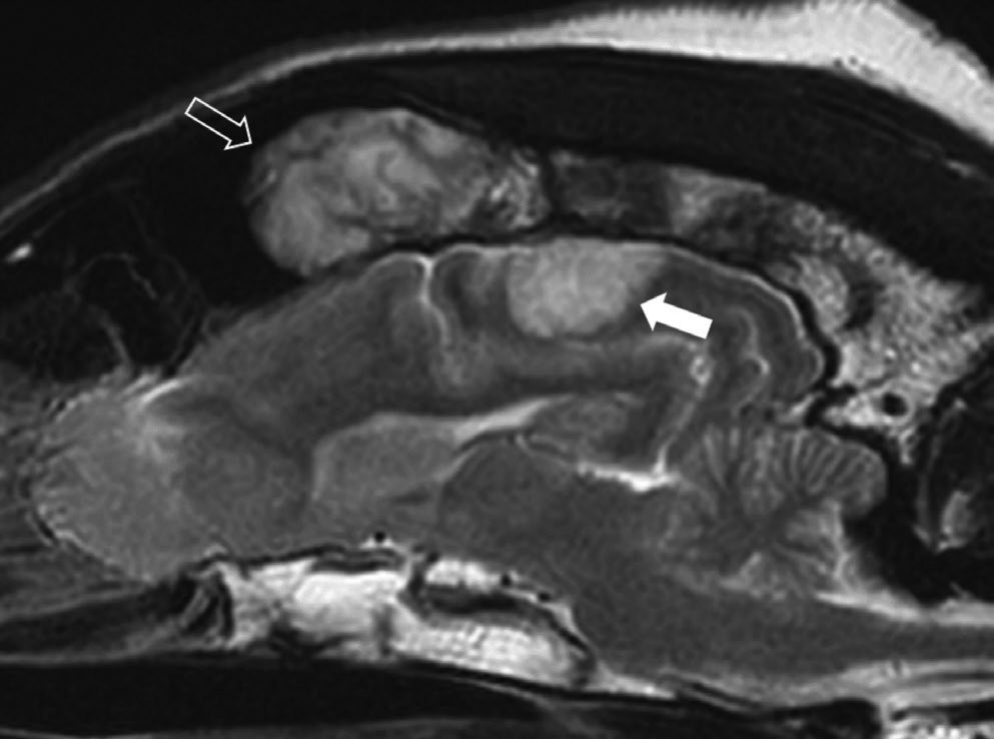
Left parasagittal T2W magnetic resonance image of the brain of the dog. A large, bulbous mass of heterogeneous signal intensity (open arrow) is present within the left frontal sinus. The infiltrative component is extending caudally into the diploe of the frontal and parietal bone. A broad‐based, well‐defined, extra‐axial, T2W‐hyperintense mass (white arrow) is noted within the parietal lobe ventral to the abnormal parietal bone

**FIGURE 2:**
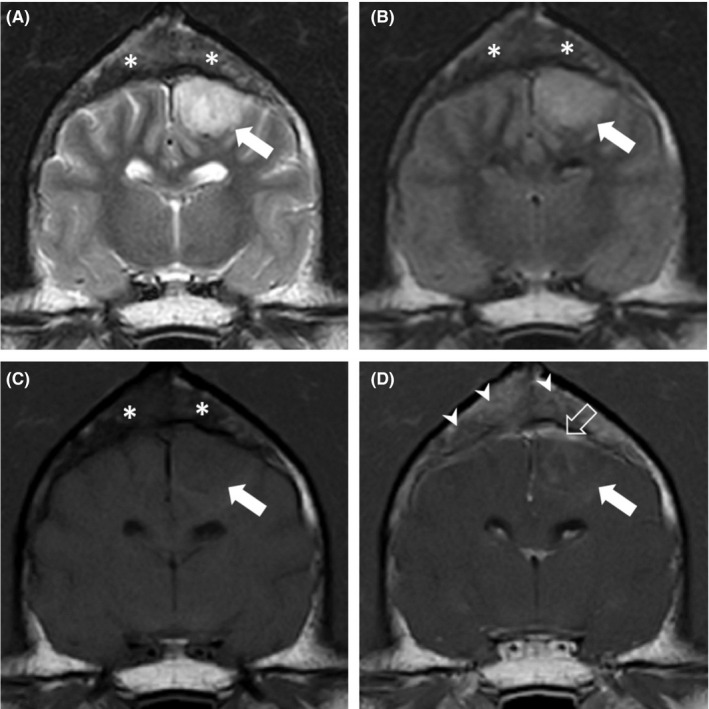
Magnetic resonance images of the brain of the dog at the level of dorsum sellae. Transverse (A) T2W image, (B) FLAIR image, (C) T1W image, and (D) postcontrast T1W image. A broad‐based, extra‐axial mass (white arrow) is noted in the left parietal lobe. It is slightly heterogeneously hyperintense on T2W and FLAIR images, but slightly hypointense on the T1W image and has a minimal mass effect. The diploe of the dorsal calvarium (asterisk) is very heterogeneous due to the loss of the normal T2W‐ and T1W‐hyperintense fat signal in multiple areas. D, The transverse T1W postcontrast image shows heterogeneous contrast enhancement of the diploe (arrowheads). The adjacent meninges are thickened and markedly contrast enhance, especially dorsal to the mass (open arrow). The mass shows slight patchy contrast enhancement (white arrow)

**FIGURE 3:**
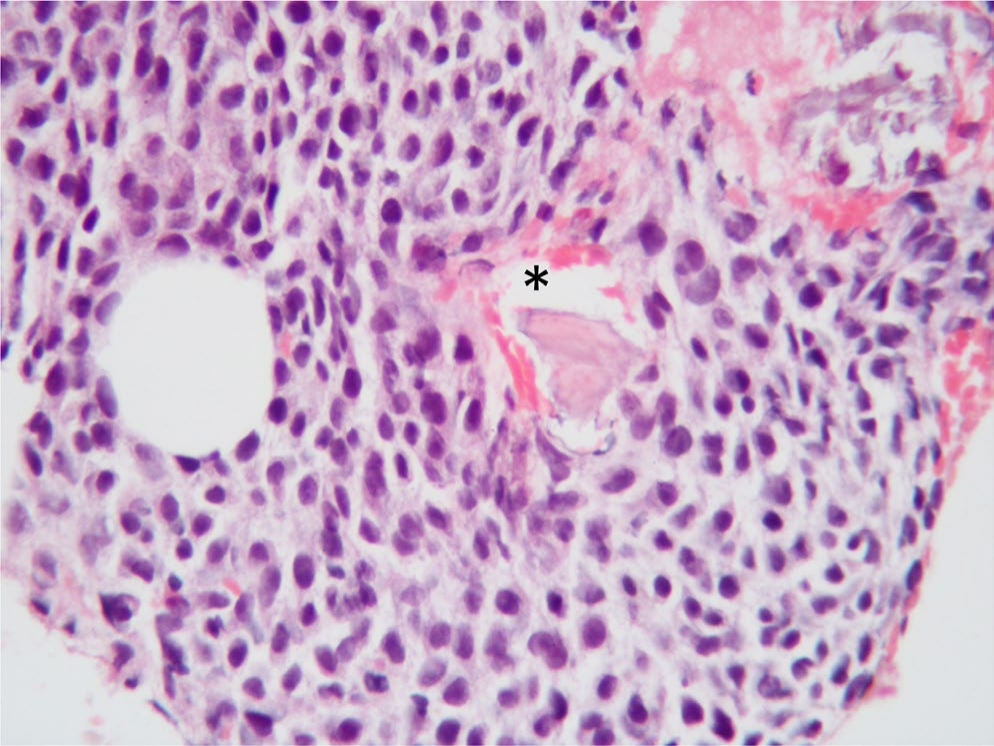
Representative histopathological photograph depicting the population of monomorphic round cells dissecting and expanding the medullary cavity. Fragments of devitalized bone trabecules are present (asterisk). Hematoxylin and eosin, ×400 magnification

Magnetic resonance imaging (MRI) of the head was performed with a 1.5 T MRI scanner (Ingenia 1.5T CX; Philips Medical System). Several sequences (T2W, T1W, T2*, FLAIR, DWI) in multiple planes were acquired. An extensive lobulated and infiltrative mass was identified centered on the frontal and parietal bones, bilaterally. Large lobules were extending into both frontal sinuses filling most of their lumina (Figure 1 open arrow). The lobules showed a marbled, mixed hyper‐/hypointensity (compared with gray matter) pattern on T2W, T1W, and FLAIR images (Figure 2A‐C asterisk) with some suspected cystic areas within the lobules with marked, also heterogeneous contrast enhancement postgadolinium injection (0.1 mmol/kg gadobutrol, Gadovist 1.0 mmol/mL; Bayer; Figure 2D arrowheads). The normal T2W‐ and T1W‐hyperintense signal (fat) of diploe was replaced by hypointense (compared with normal diploe) tissue. This tissue showed mild to moderate heterogeneous contrast enhancement. Within the internal table of the parietal and frontal bones punctate to moth‐eaten areas of lysis were present. The adjacent meninges enhanced markedly after contrast administration (Figure 2D). Additionally, another broad‐based extra‐axial mass was noted to expand from the region of left parietal bone into the left brain parietal lobe (Figure 2A‐D white arrow), displacing the cortical gray matter and compressing the adjacent gyri. The mass effect was however minimal. The mass was T2W and T2* hyperintense and did not suppress on FLAIR images. On T1W images, the mass was hypointense with a ventrolateral curvilinear hyperintense rim. The nature of this rim remained unclear. There was mild to moderate patchy central enhancement postcontrast administration (Figure 2D white arrow). The overlying meninges were markedly thickened and enhancing (Figure 2D open arrow). This mass was presumed an extension of the calvarial mass. Cerebrospinal fluid analysis was declined by the owners. A neoplastic disease of osseous origin was suspected. *Toxoplasma gondii* IgG and IgM (<50 and <20, respectively) and *Neospora caninum* IgG (<100) serum titers were not suggestive of an active infection. *Cryptococcus* spp. antigen testing was negative. Thoracic and abdominal radiographs (FCR XG‐1; FUJIFILM Medical Systems) revealed no osteolytic lesions or clinically significant abnormalities. Six weeks after presentation, incisional biopsy from the left parietal bone and underlying left intracranial mass were obtained via left rostro‐tentorial craniectomy. During the postoperative period, the dog suffered from a generalized tonic‐clonic epileptic seizure. Levetiracetam (levetiracetam 500 mg tablets; Milpharm Ltd.) (20 mg/kg PO q 8h) treatment was commenced. No postoperative neurological deficits were detected upon examination. Histopathological analysis of the skull sample revealed fragments of trabecular bone and large hematopoietic tissue. The latter was expanded by the infiltrative growth of a monomorphic population of discrete neoplastic cells associated with scant fibrovascular stroma. Cells had a scant to moderate light basophilic cytoplasm and centrally placed round vesicular nucleus with large eosinophilic nucleolus. Mitotic activity was 2‐3/high‐power field and cellular atypia was mild (Figure 3). Immunohistochemical examination indicated the presence of numerous CD79α+ and MUM‐1‐positive cells within the neoplastic population. The histolopathological examination of the subjacent intracranial tissue indicated the presence of pale eosinophilic material resembling amyloid intercalated/surrounded by small perivascular aggregates of lymphocytes. No meninges or cerebral cortex was identified in the sampled tissues.

A provisional diagnosis of solitary osseous plasmacytoma with subjacent amyloid deposition was established, and staging workup was therefore extended to exclude presence of distant tumor involvement. Radiographs of the four limbs showed no evidence of bone lesions. An ultrasound scan (RS80A; Samsung Medison Co. Ltd.) revealed normal appearance of the medial retropharyngeal, parotid, and mandibular lymph nodes, mild splenomegaly, and changes compatible with a right caudal pole renal infarct. Cytology examination of the medial retropharyngeal lymph nodes, liver, spleen, and bone marrow was performed by means of ultrasound‐guided fine needle aspirates (FNA); and no evidence of plasma cell neoplasia was detected. Serum protein electrophoresis was also performed and revealed no abnormalities.

Considering the imaging and the clinicopathological findings, a diagnosis of solitary osseous plasmacytoma with extensive calvarial involvement and subjacent intracranial amyloid formation was reached.

Meloxicam treatment was discontinued, and therapy with prednisolone (prednisolone 5 mg tablets; Millpledge Veterinary) (0.5 mg/kg PO q24 h) and melphalan (melphalan 2 mg tablets; Aspen Pharma Trading Ltd.) (3.8 mg/m^2^ PO q24 h) was instigated.

Repeat MRI of the head performed one month after diagnosis, immediately prior to radiation therapy (RT) indicated that the extra‐axial mass in the left parietal lobe was approximately 2 mm longer compared to previous MRI and had a very mild mass effect. A computed tomography (CT) scan was performed at the same time for 3D conformal radiation planning using Prowess software (Panther TPS v 5.4). The gross tumor volume (GTV) included the left parietal mass extending into both frontal sinuses and a 1 cm margin was added for planned tumor volume (PTV). Planning achieved 95% dose delivered to PTV using four 10 MV wedged photon beams (one with split wedge and one with a field in field compensation beam). Bolus tissue equivalent of 0.8 cm was used on all beams to achieve dose distribution. Both eyes and brain were outlined as organs at risk. RT was delivered under general anesthesia with a linear accelerator (Siemens ONCOR Impression Plus) using a 20 × 2.4 Gy fractionation protocol (on a Monday‐to‐Friday basis for four consecutive weeks; total dose of 48 Gy). Positioning was achieved using a thermoplastic beam directional shell (Oncology Imaging Systems) and vacuum bag (Qfix) molded to the shape of the dog's head, clipped to a head frame on a flat‐topped treatment couch, and verified using an electronic portal imaging device and megavoltage radiation.

Repeat MRI and CT scans of the head two months after completing the course of radiotherapy indicated a possible mild decrease in size of the left parietal mass and shrinkage of the right frontal sinus mass compared with previous scans although new areas of hyperostosis and sclerosis were noted in the parietal bone.

Five months after completing the course of RT, a generalized tonic‐clonic epileptic seizure was reported and the dog was re‐presented for lethargy. Abdominal ultrasound revealed an enlarged and irregular‐demarcated right kidney with a heterogeneous hypoechoic mass occupying the entire caudal pole and extending to the cranial pole. There was extension of soft tissue material within the right renal vein and the caudal vena cava. CT scan of the thorax detected multiple small soft tissue nodules throughout the pulmonary fields. The cytological examination of the renal mass was not diagnostic. These imaging findings were compatible with primary right renal neoplasia with pulmonary metastasis. Renal cell carcinoma (RCC) was the main differential diagnosis; however, other histotypes could not be excluded. The owner refused further investigations, and treatment with oral toceranib phosphate was commenced (Palladia 15 mg tablets; Zoetis) (3 mg/kg PO three times a week).

Two months later, the dog acutely collapsed and died in the household. No postmortem examination was consented by the owners.

## 2 DISCUSSION

Solitary plasmacytomas are a myeloid‐related disorder that can form either in soft tissue as extramedullary plasmacytomas or in bone as solitary osseous plasmacytomas.^1^ In dogs, solitary osseous plasmacytomas have been reported involving the axial and appendicular skeleton, as well as the zygomatic arch, and ribs.^1^, ^2^ In humans, solitary osseous plasmacytomas are most frequently seen in the thoracic vertebrae, the ribs, sternum, clavicle, scapula, or mandible.^3^ There are no reports in veterinary medicine of solitary osseous plasmacytoma affecting the cranial vault.

In the 2016 revision of the World Health Organization (WHO) classification of lymphoid neoplasms, plasma cell myeloma, solitary plasmacytoma of bone, and extraosseous plasmacytoma were classified as mature B‐cell neoplasm.^4^ Plasma cell neoplasia was excluded from the 2016 WHO classification of tumors of the central nervous system.^5^

Clinical signs associated with solitary osseous plasmacytomas usually relate to the location of the anatomic involvement. These include pain and lameness if the appendicular skeleton is affected or neurologic signs if the vertebral column is involved.^1^

In a recent review of human solitary plasmacytomas, the authors proposed that a biopsy‐proven monoclonal plasma cell infiltration of a single lesion is required for the diagnosis of solitary osseous plasmacytoma. Furthermore, there must be absence of end organ damage including hypercalcemia, renal dysfunction, and anemia. A single site of involvement without any additional lesions on imaging must be demonstrated. In addition, a diagnostic bone marrow biopsy must display <10% of monoclonal plasma cells. Lastly, immunohistochemistry staining should be performed to ascertain the presence of a monoclonal plasma cell population.^6^

In veterinary medicine, the diagnosis of solitary osseous plasmacytoma and extramedullary plasmacytoma requires tissue histology or cytology. In the case of poorly differentiated plasmacytic tumors, immunohistochemical studies, directed at detecting immunoglobulin, light and heavy chains, MM‐1/interferon regulatory factor‐4 (MUM1/IRF4), and thioflavin T, may help to differentiate from other round cell tumors. It is paramount to thoroughly stage patients with plasmacytomas which are at higher risk for systemic spread if local or loco‐regional therapy without systemic therapy is considered, especially in cases of solitary osseous plasmacytoma and gastrointestinal extramedullary plasmacytoma due to their relatively high metastatic rate. This should include bone marrow aspiration, serum electrophoresis, abdominal ultrasound scan, and skeletal survey radiographs to ensure the disease is confined to a local site prior to therapy initiation.^1^ This diagnostic workup was performed in the present case, and no evidence of distant neoplastic disease could be detected. Therefore, the conducted investigations were sufficiently thorough to support the diagnosis of solitary osseous plasmacytoma.

In the treatment of solitary osseous plasmacytomas, RT is the mainstay of therapy in humans and is commonly prescribed in pets due to the achievement of good local tumor control; however, most solitary osseous plasmacytomas in humans may progress to multiple myeloma (MM).^1^, ^6^ There is controversy as to whether systemic chemotherapy using melphalan and prednisolone improves outcome when systemic involvement is not documented.^1^, ^6^

Advanced imaging techniques (such as MRI and CT) have greatly improved the diagnosis of intracranial tumors.^7^ Nonetheless, a definitive diagnosis requires histopathological confirmation. In a study of 40 histologically confirmed intracranial tumors, the diagnosis of tumor type based on imaging findings was correct in only 70% of the cases.^8^ None of the specimens was diagnosed as plasmacytoma. In two other reviews of canine intracranial neoplasia, no description of intracranial or cranial vault plasmocytoma was provided.^7^, ^9^ Considering the rarity of the MRI findings in this case, differential diagnoses for the osseous changes included mesenchymal neoplasia (such as osteosarcoma or chondrosarcoma) and osteomyelitis; and for the parietal cortex mass, extension of neoplasia or granuloma formation was the main suspicion. Considering the extra‐axial appearance of the mass in conjunction with the superjacent osseous lesion, meningioma was also considered.^10^, ^11^

Based on the imaging diagnosis, ascertaining whether the osseous lesions were a consequence of the left parietal cortex mass or vice versa was difficult. Calvarial erosion, sclerosis, lysis, and hyperostosis associated with meningiomas, carcinomas, and glial cell tumors in companion animals and humans have been reported.^10^, ^11^, ^12^, ^13^, ^14^ Histological examination in this case suggested that the frontal parietal bone mass was the primary lesion and the underlying brain mass was amyloid deposit, presumably a complication of the plasmocytic neoplasia. Amyloidosis is histologically characterized by the extracellular deposition of insoluble fibrillar proteins. Although the nature of the amyloid deposit was not investigated further, we hypothesized this to be local deposition of immunoglobulin light chains.^15^ The presence of a local phenomenon rather than systemic amyloidosis could be due to the secretion of poorly soluble immunoglobulins with a tendency toward local precipitation.^16^ Primary local extracellular amyloidosis with secondary granulomatous inflammation is reported in plasma cell dyscrasias and plasmocytic neoplasia in animals.^17^, ^18^, ^19^, ^20^, ^21^ In the current case, no histopathological features compatible with amyloidoma (or amyloid tumor) were detected. Nonetheless, this neoplastic disease has not been reported in the veterinary literature to date. The etiology of the meningeal thickening and contrast enhancement detected on MRI was presumed to be reactive inflammation secondary to the amyloid deposit, although this remains speculative.

The lack of histopathological confirmation of the frontal sinus masses remains an intrinsic limitation of this case, although their close proximity on MRI to the left frontal/parietal bone lesion (with confirmed diagnosis) makes other differential diagnoses unlikely.

Another limitation is the lack of definitive diagnosis of the right renal growth and the multiple pulmonary nodules. Their ultrasonographic and radiological appearance was suggestive of RCC and pulmonary metastasis. Most metastasis in a study of canine RCC was pulmonary.^22^ Interestingly, 85% of dogs with metastasis at time of diagnosis had evidence of vascular invasion on histopathological examination.^22^ Another study revealed sonographic evidence of invasion into the renal vein and caudal vena cava, although surgical report did not corroborate or refute this finding.^23^ These facts may support the presumed diagnosis of RCC with neighboring vessel invasion. Moreover, the sudden collapse and death of the dog could have been associated to rupture of the invaded vessels and resultant hemoabdomen. Neurological deterioration would have been expected should the death be associated to the skull solitary osseous plasmacytoma and/or the amyloid tissue. Furthermore, plasma cell neoplasia with metastasis to the kidney or lungs has not been reported to date. The majority of solitary osseous plasmacytomas eventually progress to systemic MM in people; however, this tends to occur within months to years.^24^ To date, no documented reports of transition from solitary osseous plasmacytoma to systemic MM have been found in the veterinary literature.

## 3 CONCLUSION

Bone mass‐like lesions affecting the skull should raise the suspicion of solitary osseous plasmacytoma. Nevertheless, amyloid deposition should be considered as a possible differential diagnosis in cases of extra‐axial growths affecting the brain, when presented in conjunction with overlying skull mass‐like growths. Histopathological confirmation and thoroughly staging remain mandatory to reach a definitive diagnosis. To the authors' knowledge, this is the first description of a solitary osseous plasmacytoma with associated amyloid deposition affecting the skull of a dog.

## CONFLICT OF INTEREST

None declared.

## AUTHOR CONTRIBUTIONS

EA: contributed to the evaluation and management of the patient, collected information, and was the writer of the manuscript. DSM: contributed to the evaluation and management of the patient and reviewed the manuscript. ER: assessed the histopathology and reviewed the manuscript. FS: provided the magnetic resonance, produced the figures and their descriptions, and reviewed the manuscript. JM: contributed to the evaluation and management of the patient and reviewed the manuscript. RF: contributed to the evaluation and management of the patient and reviewed the manuscript.
